# Supplementary material for: HighALPS: ultra-high-throughput marker-gene amplicon library preparation and sequencing on the Illumina NextSeq and NovaSeq Platforms
Source: mSystems. 2026 Feb 10;11(3):e00023-26. doi: 10.1128/msystems.00023-26 (PMC13011336; doi:10.1128/msystems.00023-26)
Supplement: Supplemental Material — Supplemental figures, tables, and methodology. [file msystems.00023-26-s0001.pdf]

HighALPS: Ultra-High-Throughput Marker-Gene Amplicon Library Preparation and Sequencing on the Illumina NextSeq and NovaSeq Platforms

File S1: Methodology of the HighALPS Ultra-High-Throughput Library Preparation and Sequencing Protocol Development and Validation

(i) Primer Design Library Preparation primers

The patterned flow cells used by the Illumina NovaSeq and NextSeq series carry a higher risk of index hopping (1), and hence we chose to utilize a combinatorial unique dual-index (UDI) strategy, which enables easier detection and elimination of mismatched indices. For the HighALPS protocol we adopt the 12 nt long Golay barcodes developed by the EMP protocol (2), though shorter barcodes could also be used in this scheme. We constructed our primers for library preparation as follows:

Forward

1. 5′ Illumina adapter (AATGATACGGCGACCACCGAGATCTACACGCT)
2. Forward 12 nt Golay barcode (XXXXXXXXXXXX)
3. Forward primer pad (TATGGTAATT)
4. Forward primer linker (GT)
5. Marker gene specific forward primer (e.g. 515F: GTGYCAGCMGCCGCGGTAA)

Reverse

1. Reverse complement of 3′ Illumina adapter (CAAGCAGAAGACGGCATACGAGAT)
2. Reverse 12 nt Golay barcode (XXXXXXXXXXXX)
3. Reverse primer pad (AGTCAGCCAG)
4. Reverse primer linker (CC)
5. Marker gene specific reverse primer (e.g. 806R: GGACTACNVGGGTWTCTAAT)

The forward and reverse barcodes can be fully unique to minimize risk of index hopping, or follow a combinatorial design (whereby barcodes are re-used in different combinations) to enable multiplexing of a massive number of samples with investment in only a small number of barcodes. 96 forward and 96 reverse barcodes can be partially combined a total of 12 times per barcode to

yield 1152 unique combinations for multiplexing (Figure S1). With an estimated baseline index hopping rate of up to 2 % (1), this results in a maximum sample misassignment rate of approximately 0.04 %.

To profile bacterial communities we designed constructs that incorporate the commonly used 515F (5'-GTGYCAGCMGCCGCGGTAA-3') (3) and 806R (5'-GGACTACNVGGGTWTCTAAT-3') primer pair (4), which targets the hypervariable V4 region of the 16S rRNA gene; this is the same primer pair used in the original EMP protocol (5), but primers for other 16S rRNA genes (or other molecular targets) could be substituted here as well. For fungal ITS sequencing we designed constructs that target the ITS1 domain with the following primers: BITS (5′–ACCTGCGGARGGATCA–3′) and B58S3 (5′–GAGATCCRTTGYTRAAAGTT–3′), which demonstrate high coverage of most fungal groups (6); however, any fungal ITS primer could be substituted in this construct, in theory.

To evaluate the potential for primer dimer formation, we employed a systematic testing approach. The 96 forward barcoded primers were divided into blocks of 12, and each block was tested against a single reverse barcode construct. Primer interactions and dimerization potential were assessed using the Multiple Primer Analyzer tool (7). This analysis was repeated for each reverse barcode construct across all blocks of forward primers to ensure full coverage of potential primer pairings. Reverse barcodes that formed dimers with any forward primer block were excluded from further use. Importantly, self-dimerization of primers was not considered a primer dimerization event. A dimer-checked list of primer constructs to profile bacterial and fungal communities in a 5'-3' direction is attached in the File S2. All primer constructs were ordered in IEX-HPLC (ion-exchange high-performance liquid chromatography) purified quality, diluted and stored at -20 °C.

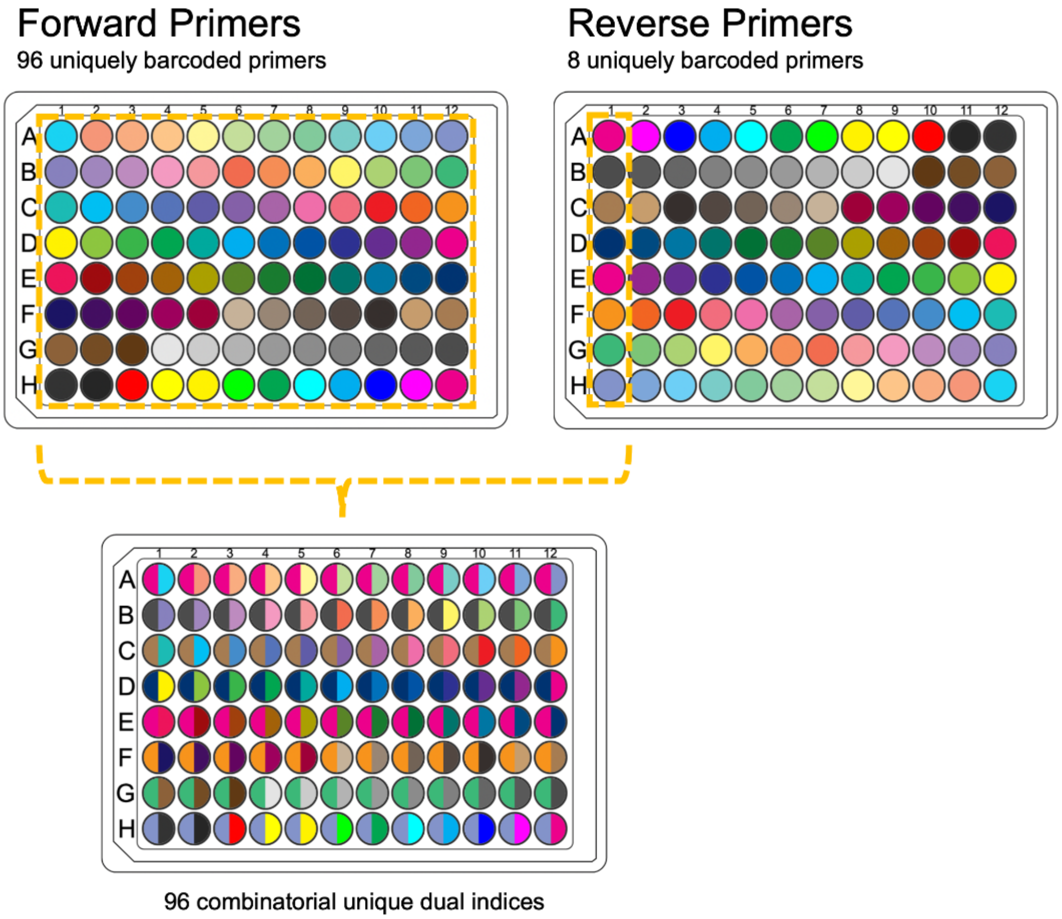

Figure S1. *Schema demonstrating the combination of barcoded primer plates for the combinatorial unique dual indexing (UDI). Using a single column of 8 unique reverse primers with 96 unique forward primers returns 96 combinatorial unique indices (indicated by the yellow frame). Repeating this with different columns of the reverse primer with a full plate of 96 forward and reverse primers respectively yields 1152 unique indices.*

Custom Sequencing Primers

A dual-indexed sequencing run on a NovaSeq or NextSeq platform utilizes the Reverse Complement Workflow (8). This process begins with (1.) sequencing of Read 1 (R1), when the R1 sequencing primer anneals to the templates during cluster generation, and is followed by (2.) Index 1 read generation for 12 cycles. Then follows the (3.) Read 2 resynthesis of the complementary strand from the original template strand, and (4.) Index 2 read for 8-10 cycles, and lastly (5.) Read 2 generations. As our library preparation primer constructs contain custom primer pads, which is where the sequencing primers bind, custom sequencing read and index primers are required (9). Additionally, since this protocol is based on a unique dual index strategy,

we require two index primers. And contrary to the MiSeq the second index reads in the newer sequencing platforms are generated after Read 2 resynthesis. The index primers therefore are the reverse complements of the read primers, i.e. index primer 1 being the reverse complement of read primer 2 and vice versa (see Figure 1). In total, this protocol requires 4 (2 read and 2 index) custom sequencing primers for each amplicon. It is noteworthy that with the sequencing chemistry (see Table 1) the annealing temperature of the platforms also changed, from 65 °C in the MiSeq to 60 °C in the NovaSeq / NextSeq (10). Illumina did not change the usage of their standard sequencing primers either, yet it is recommended that custom sequencing primer properties are as similar as possible to their standard primers (Tm = 66 °C, 33 bp, 52 % GC). The custom sequencing primer constructs for the 515/806 and BITS/B58S3 library preparation primers described above are attached in the File S3.

(ii) DNA extraction of a mock community for validation

To validate the HighALPS library preparation and sequencing protocol we used a mock community (Cat. No. D6300, Zymo Research) containing three Gram-negative bacteria that are easy to lyse (*Pseudomonas aeruginosa, Escherichia coli, Salmonella enterica*), five Gram-positive bacteria that are resistant to lysis (*Listeria monocytogenes, Bacillus subtilis, Lactobacillus fermentum, Enterococcus faecalis, Staphylococcus aureus*), and two yeasts that are also difficult to lyse (*Saccharomyces cerevisiae* and *Cryptococcus neoformans*). DNA was extracted with the MagAttract PowerSoil Pro DNA Kit (QIAGEN, Cat. No. 47109) and PowerBead Pro Plate (QIAGEN, Cat. No. 19311) according to the manufacturer's instructions. Cell lysis was performed on a Bead Mill (Retsch) and extraction on a KingFisher Flex (Thermo Fisher Scientific). Extracted DNA was stored at −20 °C until further processing.

(iii) Library preparation

Extracted DNA is amplified with a 25 µL PCR reaction with 12.5 µL of 2 X KAPA HiFi HotStart ReadyMix (Roche, Cat. No. 07958935001), 0.5 µM of each primer as well as 0.1 - 10 ng template DNA and amplified as specified in Table S1. The barcoded primer constructs are combined as shown in Figure S1 to create unique dual indices. Contrary to practice in the EMP protocol, the amplification of samples in triplicates to reduce chimera formation and jackpot effects has been shown to be unnecessary as modern bioinformatic tools are less sensitive to artifacts (11).

Depending on the quality and quantity of extracted DNA, the suggested protocol can be modified. Common issues when setting up a new library preparation protocol for a specific sample type include low yields and unspecific amplification. First and foremost, using a limited number of

cycles in PCR is highly recommended to minimize chimera formation. The number of cycles can be assessed for each sample type with a qPCR using the library preparation primers. For low nucleic acid concentration samples the proposed 1-step PCR with the barcoded primer constructs can be performed as a nested DNA. Therefore the template DNA is first enriched with universal marker gene primers, e.g. 515/806, before applying an indexing reaction with the barcoded constructs for 8-12 cycles. Further, increasing the annealing and extension time to 30 seconds can improve yield of the PCR reaction. However, excessive extension and annealing time may lead to nonspecific amplification. If the amplification of extracted DNA yields additional unspecific products, lowering the annealing temperature can increase the yield. While if only non-specific, larger PCR products are formed the annealing temperature should be increased. The KAPA Hifi HotStart ReadyMix already contains 2.5 mg MgCl2, so addition of magnesium ions to reduce mispriming is unnecessary. Primer concentration can also be adjusted to handle nonspecific amplification but should stay at a final concentration between 0.3 and 2 μM.

Table S1. *Thermocycler conditions for the amplification of template DNA with custom library prep primers.*

**Phase**	**Temperature [°C]**	**Duration [min]**	**Cycles**
Initial denaturation	95 °C	5:00	1
Denaturation	98 °C	0:30	
Annealing	e.g. 55°C for 515/806 primers, 49°C for BITS/B58S3 primers	0:15	app. 30-35 x
Extension	72 °C	0:15	
Final extension	72 °C	5:00	1
Hold	4 °C	∞	

All amplicons were purified with Agencourt AMPure XP magnetic beads (Beckman, Cat No. A63882) before being pooled equimolar and the quality of the library was checked with Qubit HS dsDNA measurement. The purity and concentration of final pools were additionally determined with a Tapestation High Sensitivity D1000 kit as well as qPCR with the standard Illumina p5 (5′-AATGATACGGCGACCACCGAGATCT-3′) and p7 (5′-CAAGCAGAAGACGGCATACGAGAT-3′) primers binding to the adapter.

(iv) Sequencing

Since the sequencing of this library requires custom sequencing primers, they have to be run on a full flow cell. For the NextSeq 2000, a Custom Primer Kit is required, as the design of the cartridge does not allow for the direct addition of custom primers to the Illumina primers, unlike the NovaSeq 6000 sequencing system. The inclusion of the PhiX Control v3 Library (PhiX) is recommended for all Illumina sequencing runs to assist with cluster mapping and template registration, with the percentage of PhiX used depending on the anticipated base diversity. For newer platforms utilizing patterned flow cells, a higher concentration of 20-35 % PhiX is advised. Notably, when including PhiX, it is necessary to combine or "spike" custom primers with the standard Illumina sequencing primers.

The pooled libraries are submitted to sequencing centers and the ultimately necessary concentration depends on the respective requirements. Apart from the pooled library, the custom sequencing primers, and list of barcodes are submitted.

(v) Data processing

Demultiplexed sequencing data was imported to qiime2 (v2024.2) (12), where reads were trimmed with cutadapt (13) (via q2-cutadapt) to remove potential remaining adapters before being denoised with DADA2 (14) (via q2-dada2). Further contaminants were removed with decontam against the negative controls (15) (via q2-quality-control). Resulting bacterial representative sequences were taxonomically classified with the 99 % SILVA 16S rRNA gene database (138 release), trimmed to the 515F-806R (V4) region (16), and fungal ASVs were classified with the UNITE database (v9.0, Version 18.07.2023) (17) with q2-feature-classifier (18) (see Figure S2). All code used in this analysis can be found here https://github.com/bokulich-publications/HighALPS-validation.

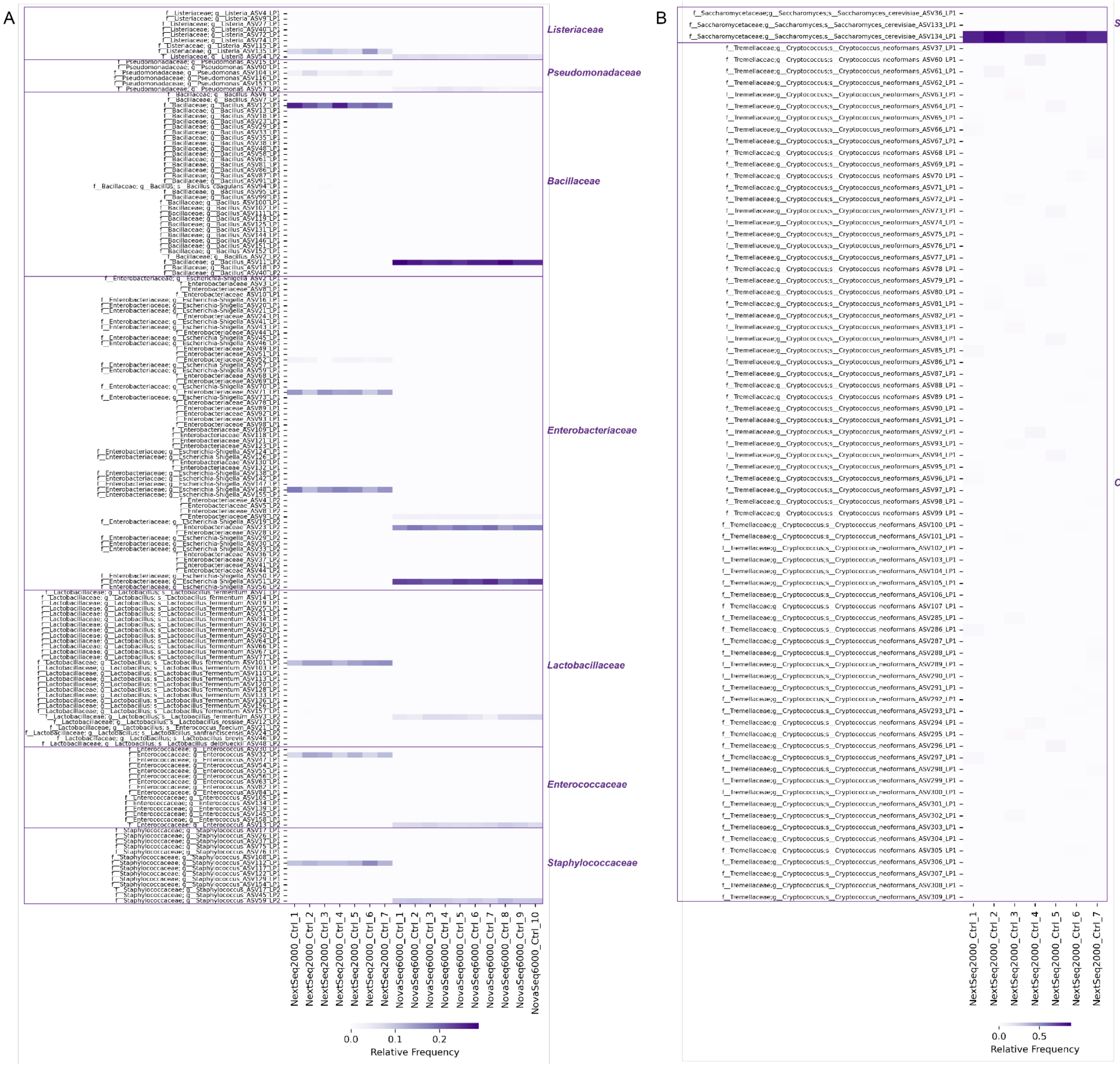

Figure S2. *Heatmap of all ASVs grouped by taxonomic family across different library preparations for (A) 16S and (B) ITS. For each expected family in the mock community, a single dominant ASV is observed per library, which is expected due to the separate denoising of the datasets. ASVs are defined by single-nucleotide differences, which may arise from genuine strain-level variation within the mock community (including differences between Zymo mock community batches) or minor sequencing errors that may be resolved, e.g., via additional quality control steps or OTU clustering at 99% (19).*

References

1. Illumina. Effects of Index Misassignment on Multiplexing and Downstream Analysis (Article #770-2017-004-D) [Internet]. 2017 [cited 2024 Sept 20]. Available from: https://emea.illumina.com/content/dam/illumina-marketing/documents/products/whitepapers/index-hopping-white-paper-770-2017-004.pdf?linkId=36607862

2. Caporaso JG, Lauber CL, Walters WA, Berg-Lyons D, Huntley J, Fierer N, et al. Ultra-high-throughput microbial community analysis on the Illumina HiSeq and MiSeq platforms. ISME J. 2012 Aug;6(8):1621–4.

3. Parada AE, Needham DM, Fuhrman JA. Every base matters: assessing small subunit rRNA primers for marine microbiomes with mock communities, time series and global field samples. Environ Microbiol. 2016 May;18(5):1403–14.

4. Apprill A, McNally S, Parsons R, Weber L. Minor revision to V4 region SSU rRNA 806R gene primer greatly increases detection of SAR11 bacterioplankton. Aquat Microb Ecol. 2015 June 4;75(2):129–37.

5. Caporaso JG, Lauber CL, Walters WA, Berg-Lyons D, Lozupone CA, Turnbaugh PJ, et al. Global patterns of 16S rRNA diversity at a depth of millions of sequences per sample. Proc Natl Acad Sci. 2011 Mar 15;108(supplement_1):4516–22.

6. Bokulich NA, Mills DA. Improved Selection of Internal Transcribed Spacer-Specific Primers Enables Quantitative, Ultra-High-Throughput Profiling of Fungal Communities. Appl Environ Microbiol. 2013 Apr 15;79(8):2519–26.

7. Multiple Primer Analyzer [Internet]. Thermo Fisher Scientific, Waltham, MA, USA; Available from: https://www.thermofisher.com/ch/en/home/brands/thermo-scientific/molecular-biology/molecular-biology-learning-center/molecular-biology-resource-library/thermo-scientific-web-tools/multiple-primer-analyzer.html

8. Illumina. Dual-Indexed Workflow on a Paired-End Flow Cell [Internet]. 2025 [cited 2025 May 30]. Report No.: 15057455 v10. Available from: https://support-docs.illumina.com/SHARE/IndexedSeq/Content/SHARE/IndexedSequencing/DualIndexWorkflowPE.htm

9. Illumina. Indexed Sequencing (#15057455 v08) [Internet]. 2020. Available from: https://support.illumina.com/content/dam/illumina-support/documents/documentation/system_documentation/miseq/indexed-sequencing-overview-guide-15057455-08.pdf

10. Illumina. Considerations when migrating non Illumina libraries between sequencing platforms (Article #1478) [Internet]. 2024 [cited 2024 Sept 20]. Available from: https://knowledge.illumina.com/library-preparation/general/library-preparation-general-reference_material-list/000001478

11. Marotz C, Sharma A, Humphrey G, Gottel N, Daum C, Gilbert JA, et al. Triplicate PCR Reactions for 16S rRNA Gene Amplicon Sequencing are Unnecessary. BioTechniques. 2019 July;67(1):29–32.

12. Bolyen E, Rideout JR, Dillon MR, Bokulich NA, Abnet CC, Al-Ghalith GA, et al. Reproducible, interactive, scalable and extensible microbiome data science using QIIME 2. Nat Biotechnol. 2019 Aug;37(8):852–7.

13. Martin M. Cutadapt removes adapter sequences from high-throughput sequencing reads. EMBnet.journal. 2011 May 2;17(1):10.

14. Callahan BJ, McMurdie PJ, Rosen MJ, Han AW, Johnson AJA, Holmes SP. DADA2: High-resolution sample inference from Illumina amplicon data. Nat Methods. 2016 July;13(7):581–3.

15. Davis NM, Proctor DM, Holmes SP, Relman DA, Callahan BJ. Simple statistical identification and removal of contaminant sequences in marker-gene and metagenomics data [Internet]. 2017. Available from: http://biorxiv.org/lookup/doi/10.1101/221499

16. Quast C, Pruesse E, Yilmaz P, Gerken J, Schweer T, Yarza P, et al. The SILVA ribosomal RNA gene database project: improved data processing and web-based tools. Nucleic Acids Res. 2012 Nov 27;41(D1):D590–6.

17. Abarenkov K, Zirk A, Piirmann T, Pöhönen R, Ivanov F, Nilsson RH, et al. UNITE QIIME release for Fungi [Internet]. UNITE Community; 2023 [cited 2024 Sept 3]. Available from: https://doi.plutof.ut.ee/doi/10.15156/BIO/2938079

18. Bokulich N, Dillon M, Bolyen E, Kaehler B, Huttley G, Caporaso J. q2-sample-classifier: machine-learning tools for microbiome classification and regression. J Open Source Softw. 2018 Oct 23;3(30):934.

19. Bokulich NA, Subramanian S, Faith JJ, Gevers D, Gordon JI, Knight R, et al. Quality-filtering vastly improves diversity estimates from Illumina amplicon sequencing. Nat Methods. 2013 Jan;10(1):57–9.

20. Thermo Fisher Scientific Inc. How pipetting choice and volume affect results of nucleic acid quantitation (Article # COL22611 0718). 2018 [cited 2024 Sept 20]; Available from: https://assets.thermofisher.com/TFS-Assets/BID/Application-Notes/pipetting-choice-volume-affect-results-nucleic-acid-quantitation-app-note.pdf

21. Illumina. Bubble products in sequencing libraries: causes, identification, and workflow recommendations (Article #1918) [Internet]. 2024 [cited 2024 Sept 26]. Available from: https://knowledge.illumina.com/library-preparation/general/library-preparation-general-reference_material-list/000001918

22. Illumina. Illumina Sequencing platforms (Article #M-GL-00451 v4.0) [Internet]. 2024 [cited 2024 Sept 20]. Available from: https://emea.illumina.com/content/dam/illumina/gcs/assembled-assets/marketing-literature/sequencing-platforms-brochure-m-gl-00451/sequencing-platforms-brochure-m-gl-00451.pdf

File S2: Detailed Step-By-Step HighALPS Protocol

The HighALPS protocol was developed for ultra-high-throughput library preparation and sequencing of microbial communities, primarily bacteria and fungi, on Illumina NovaSeq or NextSeq platforms (see Figure S3). We provide lab automation options, safe stopping points and practical tips for protocol adjustments.

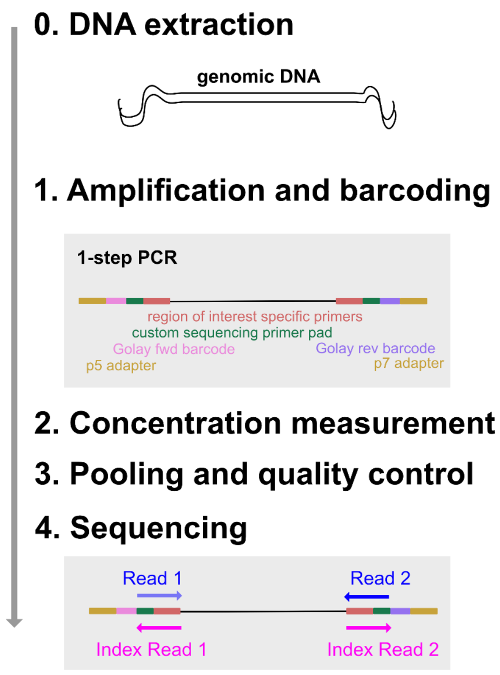

Figure S3. *Schema of the library preparation and sequencing workflow.*

Materials and Consumables

- Extracted DNA in high quality (e.g. extracted with QIAGEN MagAttract PowerSoil Pro DNA Kit Cat. No. 47109 and PowerBead Pro Plate Cat. No. 19311)
- 2x KAPA HiFi HotStart ReadyMix Polymerase (Roche, Cat. No. 07958935001)
- Forward and reverse custom library and sequencing primers as listed in Files S2 and S3, in IEX-HPLC (ion-exchange high-performance liquid chromatography) purified quality, diluted to 10 µM
- Molecular grade water
- Molecular grade ethanol
- AMPure XP bead-based DNA purification (Beckman, Cat No. A63882)

- PCR plates, 96 well (e.g. Eppendorf, Cat. No. 0030128648)
- 384 well, black, flat bottom plates (e.g. VWR-International, Cat. No. 732-3734)
- Plate sealing film (e.g. Thermo Scientific, Cat. No. AB0558)
- Filter pipet tips
- Reservoirs
- Microcentrifuge tubes, 1.5 mL (e.g. Eppendorf, Cat. No. 0030121872)
- Optional: Mock communities as positive control (e.g. Zymo Research, Cat. No. D6300)
- Qubit dsDNA High Sensitivity Assay Kit (Thermo Fisher Scientific, Cat No. Q32854)
- Tapestation High Sensitivity D1000 kit (Agilent, High Sensitivity D1000 ScreenTape Cat. No. 5067-5584 and High Sensitivity D1000 Reagents Cat. No. 5067-5585)
- qPCR dye (e.g. EvaGreen dye, 20x, Cat. No. BI-31000)
- Standard Illumina p5 (5′-AATGATACGGCGACCACCGAGATCT-3′) and p7 (5′-CAAGCAGAAGACGGCATACGAGAT-3′) primers
- Optional: KingFisher Apex 96 Standard Plate, 200µl (Thermo Scientific, Cat. No. 97002540B)
- Optional: Comb KingFisher PCR-Head (Thermo Scientific, Cat. No. 97002560)

Equipment

- -20 °C freezer
- Thermocycler (e.g., Thermo Scientific, Cat. No. A24811)
- Centrifuge with plate attachment
- Vortexer
- Pipets and multichannel pipets
- 4150 TapeStation (Agilent)
- Plate Reader (e.g. Tecan Spark 10M microplate reader)
- Magnetic stand or for automation of magnetic bead clean up optional KingFisher Apex (Thermo Fisher Scientific)
- Optional: Liquid Handling Station (e.g. Eppendorf, epMotion 5070 or Brand, Liquid Handling Station flow)

Procedure

All steps are carried out in a clean environment at room temperature unless otherwise specified.

0. Protocol optimization

Before applying this protocol it should be optimized or each sample type. The main adjustments include:

- *Cycle Number*. We strongly recommend a limited number of cycles to minimize chimera formation. This can be evaluated with a qPCR of extracted DNA and the library preparation primers (e.g. over 40 cycles with a final melting curve). Ensure the amplification curves have a sigmoidal shape and choose a cycle number for your library preparation slightly above the threshold cycle (Ct value) e.g. adding 2 cycles to the obtained Ct value. Also check the melting curve to see whether other products than your desired amplicons are formed.
- *Inhibitor check and adjust DNA input*. Nucleic acid concentration measurements can be inaccurate depending on the sample type, such as when phenolic compounds are present in extracted DNA. To mitigate potential PCR inhibition, we recommend testing varying amounts and dilutions (e.g., 10X, 100X) of input template DNA.
- *Library preparation setup*. If DNA concentrations are extremely low or of low quality, a nested (i.e. 2-step) PCR approach may become necessary.

1. Amplification

- **PCR Master Mix Calculation:** Calculate the amount of PCR master mix required (see Table S2), depending on the amount of template DNA used the volume of molecular grade water (y) is adjusted to add up to 25 µL.

Note: If performing a nested PCR to enrich the DNA template prior to tagging the amplicons with combinatorial UDI primers, the universal primers, e.g. untagged 515/806, are also added to the PCR master mix directly. A variable volume of the product from the first PCR (typically 1-5 µL) can then be used as the template for the second reaction.

Table S2. *Composition of the individual PCR reactions and calculation of a master mix for a 96-well plate. Note that when using the combinatorial UDI barcoded primers, these are added directly in combination to the respective reaction well and not in the master mix.*

**Item**	**Final concentration**	**Volume in a 25 µL reaction [µL]**	**Master Mix for 96 reactions with 10% extra**
2x KAPA HiFi HotStart ReadyMix	1 X	12.5	1325.0
Forward primer (10 µM)	0.5 µM	1.25	-
Reverse primer (10 µM)	0.5 µM	1.25	-
Water, molecular grade	adjust	$y = 10-x$	$(y * 96) * 1.1$
Template DNA (0.1-10 ng)	adjust	x	-

- **Plate Setup:** Prepare the PCR master mix on ice. Using a multi-channel pipette, aliquot (as calculated: 12.5 µL polymerase + y µL water) the master mix into a 96-well PCR plate. Add the forward primer first, followed by the reverse primer (refer to Figure S1 for guidance on combining the UDI primers), and finally, add the template DNA.

High-throughput-option: All PCR reactions can be set up using a liquid handling platform.

- **Amplification:** Seal the PCR plate securely, spin it down briefly, and place it in a thermocycler. Use the thermocycler protocol outlined in Table S3.

Table S3. *Thermocycler conditions for the amplification of template DNA with combinatorial UDI library prep primers.*

**Phase**	**Temperature [°C]**	**Duration [min]**	**Cycles**
Initial Denaturation	95 °C	5:00	1
Denaturation	98 °C	0:30	app. 30-35 x
Annealing	e.g. 55°C for 515/806 16S primers, 49°C for BITS/B58S3 ITS primers	0:15	
Extension	72 °C	0:15	

Final Extension	72 °C	5:00	1
Hold	4 °C	∞	

OPTIONAL. Magnetic bead clean up of PCR products

Before measuring obtained nucleic acid concentrations it is optional to clean the PCR products. This should be done if the PCR products are not immediately pooled and stored, as residual exonuclease activity from the polymerase might degrade the products. Or if multiple different sample types are pooled, some of which may still contain higher amounts of genomic DNA. This can be evaluated with a Tapestation chromatogram or Gel electrophoresis if larger fragments are visible.

- **Choose a bead-ratio**: The ratio is chosen depending on the expected product length. For example, using AMPure XP beads, a 0.8 X bead ratio is sufficient to preserve the amplicon and remove smaller fragments. To remove genomic DNA a two sided clean up is performed (e.g. 0.2 X first, followed by 0.8 X bead ratio).

High-throughput-option: Magnetic bead clean up can be performed on a KingFisher Apex device. We provide optimized KingFisher Apex scripts which allow bead clean up of 96 well plates within only 30 minutes (File S4).

Safe Stopping Point: The purified PCR product can be stored at -20 °C until further use but repeated freeze-thaw cycles should be avoided.

2. Quantify amplicons with Qubit dsDNA assay kit

- **Standard Dilution Series Preparation:** From the standard 1 (0 ng/µL) and standard 2 (for HS kit: 10 ng/µL), prepare a serial dilution series to cover the expected range of amplicon concentrations (e.g. 0, 0.1, 0.5, 1, 5, and 10 ng/µL). These dilutions can be stored at 4 °C for up to one month.

- **Qubit Master Mix Calculation:** Determine the total volume of Qubit master mix required (Table S4). Due to the low volume, Qubit measurements are highly sensitive to pipetting variations (20), both standards and samples should be measured in duplicates for accuracy. Further using liquid handling platforms increases accuracy.

Table S4. *For the master mix the Qubit dye is diluted with 200 X buffer. The master mix calculated for 440 reaction is sufficient for two 96 well plates as well as duplicate measurements of 6 standards.*

**Item**	**Volume in a 50 µL reaction [µL]**	**Master Mix for 440 reactions including 10% extra [µL]**
Qubit buffer	47.75	21890
Qubit dye (200X)	0.25	110
PCR product	2.00	-

- **Plate Setup:** Use 48 µL of Qubit master mix and 2 µL of amplicon or standard per well in a 384-well black, flat-bottom plate. Mix the contents thoroughly and incubate at room temperature for 2 minutes. Perform the measurements within 2 hours.

  Note. Plates should be sealed to prevent evaporation of the low volumes.

High-throughput-option: PCR products can be aliquoted and mixed with the Qubit master mix with a liquid handling platform to reduce pipetting errors and improve accuracy.

- **Fluorescence Measurement:** Measure the absorbance using a plate reader. This protocol is optimized for the Tecan Spark but can be adapted for any plate reader capable of measuring fluorescent dyes used in dsDNA quantification. The measurement parameters are the following:
  - Excitation wavelength: 485 nm
  - Emission wavelength: 535 nm
  - Gain: optimal (select well with the highest concentration of standard)
  - Z-position: from well

- **Data Analysis:** Calculate the concentration of the samples based on the standard curve generated from the serial dilutions of standards. From the concentration [ng/µL] calculate the molar concentration [nM] of each amplicon with the average library size [bp] and the average molar weight of a base pair (660 g/mol).

3. Pooling

- **Pool to equimolarity:** To achieve an equimolar pool of samples, combine amplicons in different volumes based on their concentration (e.g., 5 µL of a 10 nM library and 10 µL of a 5 nM library). Samples with very low concentrations, such as negative controls, can be spiked into the pool.

  Note. Initially, pool the samples by marker gene and perform quality control separately. Once validated, combine these into a final pool for sequencing.

High-throughput-option: Amplicons can also be pooled using a liquid handling platform to minimize human error and streamline the process.

- **Clean up:** The pools are cleaned with magnetic beads, e.g. AMPure XP beads, according to manufacturer's instructions. The bead ratio is chosen depending on the magnetic beads used and expected product length. Using AMPure XP beads, a 0.8 X bead ratio is sufficient to preserve the amplicon and remove smaller fragments.

- **Quality Control of the Pool:** Measure the concentration of the final pool using the Qubit dsDNA HS kit.

  Additionally, we highly recommend verifying the pool using a TapeStation to confirm the correct fragment size. Ideally, a single sharp peak at the expected amplicon size should be observed. There should be no smaller peaks (below ~150 bp) from primers or primer dimers after the clean up. If larger peaks from genomic DNA are visible an additional two-sided purification of the pool may be necessary. Further, a second peak at around twice the size of the expected amplicon indicates so-called bubble products which may be a result of too much DNA input or too many PCR cycles (21). Such libraries can still be sequenced but must be quantified with qPCR.

TapeStation chromatograms also allow for the quantification of the amplicon by calculating the area under the main peak. These results can be compared to Qubit measurements, noting that TapeStation concentrations are generally lower than those from Qubit.

Optional: If the concentration measurements of Qubit and TapeStation show significant discrepancies, or if bubble products are present, quantifying the final pool using the KAPA Library Quantification Kit with qPCR is highly recommended.

- **Adjusting the pool concentrations**: The target library concentration typically falls between 2 and 10 nM.

Optional Reconditioning: If pool concentrations are significantly below the target or if undesired products are present in high amounts, reconditioning can be performed using a PCR (typically only 4-6 cycles) with adaptor-specific primers.

Safe Stopping Point: Final pools should be stable at -20 °C for up to 3 weeks.

4. Sequencing

The pool, together with aliquots of the custom sequencing primers and the file of barcodes used can now be submitted to a sequencing center.

File S3: Cost comparisons of primers and sequencing platforms

S3.1. Primer Cost Comparison

Synthesizing 0.04 µmol of a library preparation primer construct of 68-75 bp length with IEX-HPLC purification costs approximately 65.12 USD / primer (based on current rates). Each unique dual index requires a forward and reverse primer and a combination of two such 96 well plates yields 1152 combinatorial UDI primer pairs. The total cost estimate per combinatorial UDI (forward and reverse) therefore is 10.85 USD (65.12 USD x 96 x 2 / 1152). Using a concentration of 0.5 µM of each primer per PCR reaction requires a total of 1.125×10^{-5} µmol primer, and the total cost therefore is 0.34 USD per 96-plate.

In contrast, a Illumina Nextera XT Index Kit v2 Set (Cat No. TG-131-2001) for 384 indices costs 1’639.37 USD (list price retrieved on 2024-09-27), which amounts to 409.55 USD per 96-plate.

S3.2. Cost comparison across different sequencing platforms and flow cells

Table S5. *Comparison of cost per gigabyte (GB) output and million reads across different Illumina platforms and relevant flow cells with list prices retrieved in Switzerland on 2024-09-27* (22) *(*considering maximum output).*

**Platform**	**Flow cell**	**Cycles**	**Cat. No.**	**Cost [CHF]**	**Output range [GB]**	**Single End Reads per Run [Million]**	**Cost per GB [CHF]***	**Cost per Million Reads [CHF]**
MiSeq	Kit v3	600	MS-102-3003	2059.00	3.8 - 15	25	137.27	82.36
NovaSeq 6000	SP	500	20028402	5077.00	65 - 800	800	6.35	6.35
NextSeq 1000/2000	P1 XLEAP-SBS	600	20100981	1676.00	10 - 60	100	27.93	16.76
